# Medical Association of Central New York

**Published:** 1910-09

**Authors:** C. R. Greenleaf

**Affiliations:** Secretary


### SOCIETY PROCEEDINGS

### Medical Association of Central New York

REPORTED BY C. R. GREENLEAF, M.D., Secretary.

THE forty-second annual meeting of the Medical Association of Central New York, held in the Masonic Temple, Auburn, New York, Tuesday, October 19, 1909, at 10 o'clock A. M.

The president, DR. M. P. CONWAY, called the association to order and spoke as follows:

Gentlemen of the Medical Association of Central New York, it is my privilege and my pleasure, on behalf of the Cayuga County Medical Society, as well as in behalf of the Auburn City Medical Society, to extend to you this morning a cordial and a welcome greeting. The committee which has had charge of the arrangement of the program has prepared such a program as will prove not only instructive but interesting to you. We here in Auburn welcome you, not only as members of the profession, but we as citizens welcome you to our city. Not long ago there were those in the Association who felt that Auburn was such a little place that the annual meetings should not even occasionally come to Auburn. The better and the more sober judgment of the members of this Association, however, decided otherwise, and I announce the opening of this forty-second annual meeting of our society by saying again that we here in Auburn and Cayuga County greet you most cordially. The Chair desires to say that the order of business has been left entirely with the Business Committee, of which Dr. Creveling of this city is chairman, and the order of business as announced by the chairman of that committee will be the order of procedure.

DR. CREVELING: The first order of business is the report of the Secretary, and we call for that.

DR. GREENLEAF, Secretary: It is moved, that the reading of the minutes of the last meeting be omitted, as they appear in the Transactions.

Motion seconded and carried.

The report of the Treasurer was called for, which was presented by Dr. Charles O. Boswell, of Rochester, Treasurer of the Association, as follows:

This report is up to last night. Received from former Treasurer, $163.61. No dues were collected after the books were handed over to me, which was in January. I have collected no dues since then, so there has been no additions to our list. The dues collected this morning will be the first I collect. Expenditures, W. H. Cornwall, Hornell, printing, on the secretary's voucher, $37.00, leaving us a balance in hand of $126.61.

It was moved and seconded that the report of the treasurer be accepted. Carried.

Dr. Creveling stated that the next order of business would be the announcement of the committees by the Chair.

The President: The chair announces that the committees are as follows, they appearing upon the program of the meeting: business; J. P. Creveling, Auburn; I. H. Levy, Syracuse; John O. Roe, Rochester. Credentials: M. M. Lucid, Cortland; A. A. Young, Newark; William M. Bemus, Jamestown. Ethics: Roswell Park, Buffalo; Henry Flood, Elmira; J. W. Eddy, Oswego. Publication: C. A. Greenleaf, Hornell; C. O. Boswell, Rochester. This publication committee, I might say in passing, heretofore has consisted of three members, but owing to the death of Dr. Krauss of Buffalo, who was, as you know, related to the official journal of this Association, the vacancy has not been filled by the chair. Entertainment: Ledra Heazlitt, William S. Cheeseman, Fred H. Parker, Louis F. O'Neill, Francis E. O'Brien, Andrew J. Forman, Sedgwick E. Austin, Sheldon Voorhees, Howard D. Chapman, Arthur H. Brown.

Dr. Creveling: It would seem proper, that the chair appoint a committee to draft some resolutions and take some action upon the death of Dr. Krauss, who has been for a number of years an active, energetic member of this Association, and the Business Committee would suggest that such a committee be appointed.

Dr. Young, Newark: I move that a committee be appointed by the chair to draw up resolutions in the matter of Dr. Krauss's death. Motion seconded and carried.

Dr. Creveling: The Business Committee finds no committees to report and no other business to be done at this time, and we therefore pass to the reading of the papers.

Dr. William M. Brown, of Rochester, read a paper entitled "Toxins and the Liver."

Discussion.

Dr. Sears, Syracuse: I simply rise to make the statement that I think the cases reported certainly show marked success, having so many convulsions and coming out right. I feel that Dr. Williams, of Baltimore, has opened up a very interesting field as to the pathology of pregnancy and eclampsia, in pointing out that the difficulty is not due to the kidney disturbance, but to the changes in the liver, and I think if we treat our cases along that line we are going to get better results. I simply rise to congratulate Dr. Brown upon the success of these cases, because they are certainly trying, and I think, regarding the one that died, that we get those cases of such overwhelming toxemia that nothing will cure them. I had a case a year ago of a woman who did a washing on Monday morning. Her physician had seen her the Thursday before. She was taken violently ill, and in spite of everything we could do she died that night at 6 o'clock. That was the most profound case I ever saw. In some of these cases we find the most profound toxemia, and I believe Dr. Williams has touched the keynote, as stated by Dr. Brown, by pointing out that the liver and not the kidney is the seat of the trouble.

Dr. Charles O. Boswell, Rochester, read a paper entitled "Some Cases treated by Vaccines."

Dr. M. M. Lucid, Cortland, read a paper entitled "Post Operative Tetanus (report of a case)."

Dr. Flaherty: This paper I think is very timely. No man who has not had a case of tetanus following surgical work can appreciate what an infection of tetanus means in a post operative case. I was unfortunate enough three or four years ago to do a rectal operation upon a case in which already there had been some rectal work done by a so-called rectal specialist, from whom I had occasion to remove some hemorrhoids. Following this operation, in three or four days, there developed tetanus and the patient died. It was a very impressive case to me, because it came like a bolt out of a clear sky. As a result of that case I looked up some literature to see how frequently this condition occurred. In a very interesting paper read in 1906 I think Dr. Jacobson reported five or six cases of this kind. I was able at that time in the literature at my command to find several cases that had followed. Since that time a number of cases have been reported. The idea Dr. Jacobson expressed in his paper at that time was that these infections did not come from without, but that they were due to the tetanus bacilli which was already in the intestinal tract, and that the infection came from the contamination in some way of the intestinal contents.

As I say, at that time there had been a number of cases reported of tetanus following aseptic surgery appearing in rectal work, and that would go to bear out the idea that the tetanus bacillus is in a certain number of instances a normal happening of the alimentary tract. The idea that Dr. Matas brought out a year ago, and which Dr. Lucid has expanded upon today, that the tetanus bacillus came to the intestinal tract through the eating of raw vegetables, I think is a very good explanation of the occurrence of tetanus in this way. The doctor goes still further; besides giving the antitoxin in all of his rectal work, he withholds raw vegetables from his patient, and I think it is a mighty valuable thought to bear in mind. While tetanus does not occur so often, still, one case in 15,000 is well worth saving. After the thing has once developed it is nearly impossible to save the patient, but the withholding of the raw vegetables and the giving of the antitoxin in all these cases in which there is any possibility of getting into the intestinal tract, it seems to me is the one good point brought out.

DR. JACOBSON, Syracuse: It was my privilege to be asked by the officers of the American Surgical Association three years ago to present to them certain observations regarding tetanus cases. I associated with me Dr. Pease, of Albany, who had made personal investigation of the pathological side of tetanus, and had his valuable coöperation. We went over this work very fully, and I refer you to the account of that work in the *Annals of Surgery,* following that meeting in 1906. I also had the privilege of listening to Dr. Matas at the last meeting of the society. In reviewing this work I was very much impressed with the question of post-operative tetanus, and the interesting feature was that in almost all the cases published it has occurred in clean cases, and none of the cases to which I referred at that time were rectal cases.

The only rectal cases I have ever seen was the one to which Dr. Flaherty referred, and on which I was with him in consultation. The interesting feature of that case was that it had been treated by a so-called rectal specialist, who had left a nasty condition, and then after the case had come into Dr. Flaherty's hands in a septic condition, this condition of post operative tetanus appeared. The early features of that case were instructive and bore out some of the points in the paper I refer to, namely—as to the early local manifestations. There Dr. Flaherty will recall the early clonic indication was the rectal spasm, and for the first twenty-four hours we were very much in doubt whether these were rectal spasms or not. In the end, however, the course of the disease bore out the fact that these rectal spasms were the earliest manifestations of the germ of tetanus. Now, that is the only case in which I have seen a condition which was associated with the intestinal tract in which we have had tetanus.

It is a common thing for us to see after the dirt infection, the tetanus infection being a dirt germ, but in the paper to which I referred most of the cases in which we have post operative tetanus were following operations for hernia and appendicitis. Both of those, of course, concern the intestinal tract. They were all of them clean cases of appendicitis. The same thing was true of the hernias. The hernias were not operated upon during a period of strangulation. Then there were some cases reported in which tetanus had followed hysterectomy. We want to bear one thing in mind, and that is, you cannot always trace the course of the tetanus infection. We want to remember that the material which we use for ligature and suture work is an intestinal product, being taken from the gut of the sheep. Catgut is a constant menace and a source of disturbance, and we are not always able with a central incision into the abdominal cavity, in a case of tubercular peritonitis, to avoid infection, for by simply opening the abdominal cavity you have established a communication with the contents of the intestine, and in the case before us, that the danger, or the actual area of infection, was from the intestinal tract.

We want to remember that here was a case in which catgut was used, and while post operative tetanus does occur only rarely, I believe in practically all the cases in which it has occurred catgut has been used for ligature and suture work, and while catgut is unquestionably free of the tetanus bacillus in most instances, I believe every now and then we run across a part of a strand in which there may be nesting a tetanus germ, and I am more inclined to that belief than that it came from the contents of the intestine, because we have all of us done so many abdominal operations and we do not get any tetanus from such work. In more than thirty years of surgical work I have never seen a case of tetanus following this condition. Another thing, the large intestine is, so to speak, a cesspool. It receives the sewage of the whole tract, and all its material loaded with bacteria. However, there is a great deal more about tetanus we don't know than we do. In regard to the point Dr. Lucid brought out in his paper as to the reports of tetanus and the treatment of antitoxin, in my paper I avoided all published work in literature and wrote to all the hospitals of the United States that had much surgical work, asking them to send to me from their hospital records the tetanus cases they had as a result of infection in operative work, and the paper to which Dr. Flaherty refers in its result is not based upon any haphazard publication, but upon the complete publication of the surgical treatment of the largest hospitals of the United States, and it was found that where tetanus antitoxin was applied, in no matter what form, whether injected into the brain, into the nerves, into the blood subcutaneously, or into the muscles—and I have a series of tables covering each of these—it did not seem to make much difference if tetanus was once established, and the mortality in acute tetanus—and I make two classifications, those occurring in the first week and those after the first week of exposure—it did not seem to make much difference as to how you injected it, or in what quantities you injected the antitoxin, something like 88 per cent. of all cases died. Now, on the other hand, in all of the hospitals that have been practising the prophylactic use of antitoxin, in all cases where there was dirt infection or stable infection, or in anyway where the wounds had come in contact with manure or any of the filth that might be a tetanus bacillus carrier, practically never a case of tetanus. The immunising dose was sufficient in practically every case to prevent the occurrence of tetanus.

Every now and then a case would be reported of late tetanus following the prophylactic use of antitoxin, and when it did occur it occurred in very mild form. I think the important thing is this, and the rule we have followed in our surgical work, that in all cases of dirt infection use tetanus antitoxin. I cannot say that I am ready to affirm that in all cases of operative work you have got to give every person an immunising dose of tetanus antitoxin; that is a question for the future to evolve, as to just where the infection comes from. Every now and then you stir up an infection after vaccination or after burns. We found a number of cases of tetanus that followed vaccination, and came long after the inoculation, after vaccination to prevent smallpox, and came when there was an open wound to be contaminated. We also found in cases of burns that we don't get the tetanus until weeks after the burns, and then when it appeared it was contrary to the rule of a mild course, but in almost every case of burns, no matter if the infection was three or four weeks after, when tetanus occurred it was furious and past killing. I say, there are a lot of things which concern this matter which we cannot figure out, which I believe it is a matter we will become more familiar with and know more about in the future. However, I am very much pleased to have this paper presented, and even if it is only for the purpose of emphasising the importance of using tetanus antitoxin as a prophylactic and not expecting it is going to do much good when the disease has already been established.

Dr. Howe, Phelps: This able paper of Dr. Lucid and its discussion has appealed to my interest two-fold. First, on account of its importance, and second, on account of my relationship to the State Department of Health, in which I am privileged to watch the results obtained in the use of the antitoxin, and it is that question in particular to which I desire to speak for a moment. I note with a great deal of pleasure of the universal recommendation of the antitoxin in immunising doses. I am sure the department will be greatly gratified, so far as being a remedy prophylactic, with this aspect. The department I am sure, gentlemen, will welcome all suggestions from the practising physicians of the state as to the action of the antitoxin as a remedy per se in the treatment of acute cases. If the antitoxin is not of value in acute cases the department will, I am sure, be desirous to ascertain the reason why. Still further, gentlemen, the law requires that your local health officer shall keep on hand at all times a fresh supply of antitoxin, to which physicians have access in poor cases, and the department never draws the line, and you are practically to be left to use it on all cases; so I would ask that all physicians make certain that their local health officers are keeping in stock a fresh supply of antitoxin. That may also be the reason why antitoxin is not efficient as a remedy. You may use some old antitoxin that has lost its force; so the department would like to ask that you physicians see that your local health officers, watch their antitoxin supply. If it is at all aged it is an easy matter to supply it. I am very much gratified with the results that are obtained.

Dr. Voorhees: I have in mind a case in which the symptoms developed in a young unmarried woman. These symptoms of tetanus developed without any injury, so far as could be traced, no operation of any sort, and they began in the evening after a no more than ordinary day of work. I saw her in the early evening, with high temperature, rapid pulse, and the other symptoms were regularly defined. I again saw her early the next morning. The symptoms had been developing during the night, with well marked convulsive seizures occurring about the mouth. The antitoxin was then administered about 12 o'clock of the day following its beginning, after the diagnosis of tetanus. The patient failed rapidly from the first. The seizures became more and more frequent, and the patient died about 30 hours after the first appearance of tetanus. This was a case developing without injury or a prophylactic procedure.

Dr. Lucid: I just rise to express my appreciation of the interest taken in this paper, and I am more than pleased to know we have a representative of the State Department here. I would like to offer this suggestion, that if this antitoxin was placed not only in the hands of the health officers but in the various hospitals throughout the state it would be a great advantage many times. It is often difficult to obtain it fresh from the health officer, and we would feel safer if we knew this was kept in the hospitals. I offer this simply as a suggestion to our State Department,—if this could be kept in the various hospitals and kept on hand continually, rather than to depend entirely upon the local health officer.

Dr. Voorhees: I would like to know how old antitoxin can be and still be considered fresh.

Dr. Robert T. Morris, New York: As a guest I do not like to intrude in this discussion.

The President: We are very glad to have you speak, Dr. Morris.

Dr. Morris: Apropos of this question, I would like to make one point. The only case of tetanus I have lost for a great many years occurred in a student at Cornell University, an athletic man, in perfect health. I operated upon him for hernia. He developed tetanus about a week after the operation. The wound had healed by primary union. The tetanus bacillus was found. We telegraphed to New York for a supply of tetanus antitoxin. That was on Friday. The next day was a legal holiday, and Sunday followed, and no antitoxin could reach that man, although if we had had antitoxin on hand at Ithaca at the moment when the symptoms were recognised in that case there is a possibility, or probability, that we might have done very much for that patient. The man died.

Dr. Creveling: Called for the reading of the paper, "Some Desires of the State Department of Health," by Dr. William A. Howe, of Phelps, Director of the Division of Communicable Diseases.

Dr. Howe: Before presenting my paper I will say in reply to Dr. Lucid's suggestion that I think it is indeed a wise one. I can see no reason why tetanus antitoxin and diphtheria antitoxin should not be in the hands of all hospitals of the state, and I will say to you as a new member of the department, because I have only been identified with the department a very short time, that I will endeavor to see that such is accomplished. The case of which Dr. Morris speaks is another illustration of the actual neglect which many of our health officers show, and I say that, conscious of the fact that I have been guilty myself many times of the same thing,—to see that the preparations supplied by the State Department are fresh and in abundance. The State of New York, does not purpose to be stingy nor penny wise in its support to the profession nor in its effort to avoid disease, and I am sure that Dr. Porter will welcome all kinds of wise suggestions and criticisms from the profession leading to the improvement of the service of the department; in fact, that is my function here today, to appeal to the profession of the state for its more active coöperation with the department, and to assure the profession that the department is desirous of acting with the profession.

As to the age of the antitoxin, as to when it loses its efficiency, I must say to that speaker, as I have always admitted in my practice, that if I don't know I propose to say so. Now, I am new in this line of work, but there is another question which I will take up at once. The instructions, as you will find on the circular accompanying these antitoxins, states that so soon as the solution appears at all cloudy that it should not be used, it should be returned at once for a fresh supply. That is the rule which I have used in my practice, but to me it would seem as though if any of those antitoxins were a year old they should be returned. I do not believe it is wise, especially in a condition as vitally important as tetanus, where it is absolutely necessary for the efficiency of your treatment that you use an early immunising dose—I say it is essential that you should have a fresh antitoxin as a remedy.

Now, before reading my paper, which will be brief, I am desirous, in behalf of the State Commissioner of Health, of inviting the members of this association, and through you the physicians of the state, to attend the annual conference of health officers which will be held in Rochester on the 10, 11 and 12 of November. It is our purpose at that time to hold a three days' session in the Convention Hall at Rochester. The program promises to be one of the most interesting, and, I think, instructive, held by this association. It is expected that we will have from twelve to fifteen hundred health officers and physicians present. We would like to see even more than that.

Dr. Howe then read the paper entitled "Some Desires of the State Department of Health."

It was moved and seconded that the Association take a recess until 2 o'clock. Carried.

Afternoon Session, 2.40 o'clock.

The president announced as the first order of business the election of a president and vice-president for the ensuing term.

Dr. Mercer: I would like to nominate Dr. Sears, of Syracuse, for the Presidency. Seconded.

Dr. Jacobson: It has always been the custom of this Association to elevate to the presidency the vice-president. I take pleasure, therefore, in placing before this society the name of Dr. Stephenson for the presidency of the Association.

Dr. Crego: I would like to second the nomination of Dr. Stephenson, being the first vice-president, and I sincerely hope he will be elected.

The nomination of Dr. Sears was seconded.

Dr. Wynkoop seconded the nomination of Dr. Stephenson.

There being no further nominations the president declared the nominations closed.

The president appointed as tellers Dr. Wynkoop and Dr. Flaherty.

While the ballots were being taken and counted for the office of president, the president called for nominations for the office of vice-president.

Dr. Crego, of Buffalo, was nominated. Nomination seconded.

Dr. Greenleaf: The next meeting will be held at Rochester, and it has been customary to name the vice-president from the city where the next meeting is to be held. I therefore nominate Dr. Wesley T. Mulligan of Rochester for vice-president.

Dr. Crego: I would like to second that nomination and decline for myself the nomination for first vice-president.

The President: Dr. Crego has declined the nomination; are there any other nominations? If there be no objection the chair will declare the nominations closed, and suggest that the tellers cast the ballot of the Association for Dr. Mulligan for the office of vice-president.

Motion made and seconded that the secretary cast the ballot of the Association for Dr. Wesley T. Mulligan of Rochester, for first vice-president. After the secretary complied the president declared him duly elected for the ensuing term.

The Secretary: The ballot for president has been declared by the tellers to be a tie, thirty-eight each.

The President: There being no election, the Association will ballot again for the office of president.

Dr. Crego: I would like to inquire if it is the rule in this association to elevate the vice-president to the presidency, if it is a time honored custom?

Dr. Jacobson: I can answer that by saying there has been very few exceptions in the history of this society when that has not been done. I was informed just now there was one exception, but it has been so universally the rule that it has been practically the time honored custom to take that stand.

The President: While the tellers are counting the vote, the chair wishes to announce that Dr. Crego, of Buffalo; Dr. Craig, of Eldridge, and Dr. O'Brien, of Auburn, have been named as a committee to act upon the motion in regard to the death of Dr. Krauss of Buffalo.

The tellers reported the result of the second ballot for president as follows:

Dr. Sears received 42 votes.

Dr. Stephenson received 38 votes.

Dr. Stephenson: I move that the vote he declared unanimous. Seconded and carried.

The president declared Dr. Sears duly elected president for the ensuing term.

The president called for the report of the Committee on Credentials.

Dr. Lucid: Mr. President, the treasurer's report, the books show there are two names, Dr. John J. Baker of Syracuse and Dr. Eddy of Geneva, eligible for membership in this Association.

Dr. Creveling: I move that as soon as the men named comply with the requirements of the constitution they be recorded as members of the association. Seconded and carried.

(*Concluded in October*).

